# Progress in Medicine

**Published:** 1897-03-06

**Authors:** 


### Progress in Medicine.

### ANÆMIAS.

**Chlorosis.**—The discussion at Carlisle last summer cannot be said to have brought forward anything new on the cause of this disease. The treatment by rest, purgatives, iron, and intestinal antiseptics were acknowledged as effective, though Bezly Thorne advocated in addition the use of the Schott methods. Since then, Fedeli[1] has reported on the use of ovarian extract, which gave valuable results. He believes the disease to be a neurosis acting through changes in the ovarian secretion baout puberty. The ovary, he thinks, acts as a hæmatopoietic both reflexly and by means of an internal secretion, while the use of ovarin causes a true polycythæmia and increase in hæmoglobin with general improvement. Ch. W. Townsend[2] treated a series of cases with (*a*) beta-naphthol for intestinal antisepsis, producing an average gain of hæmoglobin of 1·8 per cent. per week; (*b*) with iron, where the gain was 5 per cent. per week; (*c*) about a third of those treated with iron had received naphthol previously, and here the gain was 6·7 per cent. Others were treated with iron and naphthol together, and here also a higher figure (7·9) was reached than when either drug was used alone; (*d*) one group was treated by cascara alone, and here there was an actual loss of hæmoglobin. D. J. Leech[3] gives a useful account of the controversy whether iron is or is not absorbed from the intestinal tract, and the experiments of Hall, Schmiedeberg, Quincke, Gaule, and others on the subject. Hall found that iron, at any rate in the form of carniferrin, is absorbed by the duodenum. Some of it is converted into hæmoglobin, and some stored up in the spleen and liver. Macallum proved that various forms of iron could be absorbed throughout the entire intestine, and could be detected in the adjoining vessels. Opinions differ as to the form in which the iron is absorbed—whether as an albuminate or a carbohydrate of iron—but it is clear that much is excreted by the large intestine. Dieballa[4] describes a rare complication of chlorosis, papillo-retinitis, in a girl of twenty-one. In March the sight became defective, and in April double optic neuritis was found, with slight paresis of the left abducens nerve. The papilla was greyish-red, swollen, and blurred round the edge, the veins were wide, the arteries narrow, and the vessels hidden in places. The hæmoglobin stood at 31, and the red cells numbered 3,880,000. In September she had recovered perfectly. The onset was preceded by over-exertion and menstruation, and marked by severe headache, which disappeared under rest and iron.

**Splenic Anæmia** is, according to F. Taylor,[5] probably not so rare as has been thought. Cases have been recently described by himself, by Dr. S. O. West, and Dr. Shaw. He finds the first symptom is pain from the enlargement of the spleen, followed by anæmia with excessive diminution of hæmoglobin. Hæmorrhages are less common than in leucæmia, sometimes there is pyrexia, and at a late stage nausea, vomiting, and great emaciation, ending in death. The spleen shows a sclerosis of the Malpighian bodies and fibrous thickening of the trabeculæ. In West's case there was no increase of iron in the spleen or liver, but in Dr. Shaw's this was very marked. The causation is very obscure. Some improvement followed the use in Taylor's case of oxygen, 30 litres daily, with arsenic and iodide of potash. Splenectomy is also suggested. Though its fatal results in leucocythæmia are well known, excision in other diseases is followed by improvement of any anæmia present, and in clinically doubtful cases splenic anæmia is easily distinguished from leucæmia by examination of the blood.

**Pernicious Anæmia.**—Aug. E. Eshner and Wharton Sinkler[6] report three cases of this disease in one family —a father and two daughters. The father was attacked four years before the daughters, and died with symptoms of posterior spinal sclerosis, which varied with the amount of anæmia. A complete histological examination of the cord, however, could not be made. Whether there was any cause for the disease common to all three patients was not discovered; but the coincidence is of more than ordinary interest. All were hardworking, fairly temperate people, and the girls seem to have had insufficient food. R. E. Cabot[7] studied the pathology in fifty cases, and found that deformed blood cells are not more numerous than in other forms of anæmia; the commonest change is the absence of the central bi-concavity, but variations in size above and below the normal are present in 90 per cent. of the cases. As well as nucleated red cells, certain cells were found also that stained in an abnormal manner. Ruttan and Adami[8] made an analysis of a large quantity of blood in this disease. The serum gave a specific gravity of 1026·1, or rather less than the normal, the proteids were deficient 40 per cent., the globulins also abnormal, and the ash 12·5 per cent. above normal. In the liver there was an excess of iron, as W. Hunter has noticed in other cases. Stein[9] discusses the functions of the megaloblasts which are peculiar to the blood of pernicious anæmia, and regards them as embryonic cells having great regenerative power, appearing only in this form of anæmia, and implying an extraordinary effort of the organism to restore the marrow to a healthy condition. However, the true pathology and causation remain obscure, Hunter insisting that the disease is a definite one, depending on a toxic destruction of the blood in the portal circulation and deposition of the iron in the liver; while Stockman regards it as merely a high degree of anæmia produced by frequent small hæmorrhages. The cord changes referred to in Sinkler's case, mentioned above, have been investigated amongst others by C. Eugene Riggs[10] in the case of a woman aged forty-five. The degeneration affected the anterior pyramidal, the direct cerebellar, the crossed pyramidal and Lissaner's tracts, as well as the posterior columns. It extended from the first cervical to the fifth lumbar segments, and was not related to the extravasation of blood which occurred in the mid-dorsal region. The latter symptom has been also noticed by Petren[11] with patches of sclerosis caused by it. Even when no clinical symptoms appeared there was degeneration of the posterior columns; but other parts were also affected. Petren believes that a toxic process underlies some at least of the changes. As to treatment, injections of sublimate 5 millegrammes daily seem to have produced a cure in a case reported by Paterson.[12] An American author[13] advises treatment with salol and beta-naphthol if the case is thought to depend on intestinal intoxication, and quotes successful instances of their use; whereas if the blood-forming organs are defective (which is not an acknowledged cause in this country) he prefers marrow, arsenic, or oxygen. Nothing has been discovered as to the mode in which marrow brings about the changes which have been accredited to it. J. Michell Clarke[14] and others have noticed the occurrence in syphilis of a state indistinguishable from the blood changes in pernicious anæmia, and relieved by specific remedies. One remarkable cure by raw marrow, after iron and arsenic had failed, is recorded by Blumenau[15] in the case of a soldier who was attacked by the disease after severe dysentery, while Dieballa succeeded in like manner with salol.

**Leucocythæmia.**—A case of the rare and rapidly fatal lymphatic type is described by J. Craig.[16] The disease ended fatallyin two months. There was general lymphatic enlargement, with dyspnœa, epistaxis, and profuse diarrhœa. The temperature varied from 99 deg. to 103 deg., and the white corpuscles were increased to the amount of one to twenty red. This increase was practically confined to the small lymphocytes, though the spleen and liver were enlarged, as well as the other glands. This, of course, is the form nearest allied to Hodgkin's disease, and another case is detailed by C. F. Martin and Mathewson,[17] of Montreal. The blood showed here a proportion of white cells one in seventy-five and upwards. The attacks followed an affection of the lips and gums, and the authors remark on the number of cases in which some intestinal affection has preceded the disease, suggesting that a toxic agent has entered by this channel. On the question of the diagnosis of leucocythæmia from Hodgkin's disease they show that all the diagnostic tests fail in special cases, both in acute and chronic cases; that the leucocytosis in the former disease is inconstant and may be imitated in the latter. Even the special forms of cells are not strictly confined to either. Thus, if their arguments are accepted, we are unable to advance beyond the old diathèse lymphogène, as every test for leucocythæmia can be found in undoubted cases of malignant and other secondary anæmias.

Gumprecht[18] finds that in the few cases of leucocythæmia, where the uric acid is not increased, the alloxur bodies, or those containing xanthin or nuclein bases, are increased, and that this varies with the amount of the leucocytosis. This gives a fresh argument in favour of the derivation of uric acid from the destruction of leucocytes.

The leucocytosis produced by the administration of nuclein is, according to W. A. Wells,[19] only a local condition, a determination of the white cells into the peripheral circulation. They are not new ones, for at first all cells are mononuclear. The polynuclear are the most sensitive to chemotaxic influences, are easily caused to migrate, and play the chief part in the protection of the body.

S. Moraczewska[20] shows that in pernicious anæmia the alkalinity of the blood is very low, the specific gravity very high, and the red corpuscles contain an excess of colouring matter. In chlorosis the alkalinity is low, the specific gravity high, and the corpuscles contain little hæmoglobin; but in carcinoma the alkalinity is high, the gravity diminished, and the corpuscles contain little colouring matter. Jacob has recently denied that any increase of alkalinity takes place in animals where an artificial leucocytosis has been produced.

An attempt to give an exhaustive analysis of the blood changes in leucocythæmia is made by R. J. M. Buchanan.[21] He notices in the splenomyelogenous form, not only myelocytes, but an increase in the coarsely granular oxyphile cells, blood plates, and fibrin; in the lymphatic type there is less leucocytosis, and an increase in the lymphocytes; and in Hodgkin's disease there may be a slight increase of finely granular oxyphile cells. Twenty-two coloured figures, exquisitely executed, of the various cells discussed, elucidate his propositions. Rolleston[22] points out the difference, not only between leucocythæmia and Hodgkin's disease in its soft or hard form, but also between the latter and lympho-sarcoma. The latter is strictly only a pathological term, but generalised sarcoma may be difficult to distinguish from lymphadenoma. Besides the frequent improvement of the latter under arsenic, it is important to note that the growth does not infiltrate the tissues around, or spread out of the glands affected. In his experience the "hardbake" spleen, showing white masses on a pink ground, is not common in cases of this disease.

[1] B. Med. Journ., Dec. 5. [2] Med. Chron., Nov. [3] Med. Chron., Nov. [4] B. Med. Journ., Oct. 17. [5] B. Med. Journ., Sept. 19. [6] Amer. J. Med. Sciences, Sept. [7] Lancet, Aug. 29. [8] B. Med. Journ., Dec. 12. [9] B. Med. Jour., Oct. 10. [10] Inter. Med. Magaz., Sept. [11] Lancet, Sept. 19. [12] B. Med. Jour., July 18. [13] Med. Record, Nov. 14. [14] B. Med. Jour., Sept. 19. [15] Med. Week, Oct. 16. [16] Dublin Jour. Med. Science, Sept. [17] B. Med. Jour, Dec. 5. [18] B. Med. Journ., Dec. 26. [19] Med. News, Oct. 17. [20] Amer. Med. Surg. Bulletin, Oct. 17. [21] Jour. Path. and Bact., Dec. [22] Clinic Jour., Oct. 28.

